# Pregnancy outcomes in facility deliveries in Kenya and Uganda: A large cross-sectional analysis of maternity registers illuminating opportunities for mortality prevention

**DOI:** 10.1371/journal.pone.0233845

**Published:** 2020-06-01

**Authors:** Peter Waiswa, Brennan V. Higgins, Paul Mubiri, Leah Kirumbi, Elizabeth Butrick, Rikita Merai, Nancy L. Sloan, Dilys Walker, and the Preterm Birth Initiative Kenya & Uganda Implementation Research Collaborative

**Affiliations:** 1 Maternal, Newborn and Child Health Centre of Excellence, School of Public Health, College of Health Sciences, Makerere University, Kampala, Uganda; 2 Department of Public Health Sciences, Karolinska Institutet, Stockholm, Sweden; 3 Department of Pediatrics, University of California San Francisco, San Francisco, California, United States of America; 4 Centre for Clinical Research, Kenya Medical Research Institute, Nairobi, Kenya; 5 Institute of Global Health Sciences, University of California San Francisco, San Francisco, California, United States of America; 6 School of Medicine and Department of Obstetrics-Gynecology and Reproductive Sciences, University of California San Francisco, San Francisco, California, United States of America; Johns Hopkins School of Public Health, UNITED STATES

## Abstract

**Introduction:**

As facility-based deliveries increase globally, maternity registers offer a promising way of documenting pregnancy outcomes and understanding opportunities for perinatal mortality prevention. This study aims to contribute to global quality improvement efforts by characterizing facility-based pregnancy outcomes in Kenya and Uganda including maternal, neonatal, and fetal outcomes at the time of delivery and neonatal discharge outcomes using strengthened maternity registers.

**Methods:**

Cross sectional data were collected from strengthened maternity registers at 23 facilities over 18 months. Data strengthening efforts included provision of supplies, training on standard indicator definitions, and monthly feedback on completeness. Pregnancy outcomes were classified as live births, early stillbirths, late stillbirths, or spontaneous abortions according to birth weight or gestational age. Discharge outcomes were assessed for all live births. Outcomes were assessed by country and by infant, maternal, and facility characteristics. Maternal mortality was also examined.

**Results:**

Among 50,981 deliveries, 91.3% were live born and, of those, 1.6% died before discharge. An additional 0.5% of deliveries were early stillbirths, 3.6% late stillbirths, and 4.7% spontaneous abortions. There were 64 documented maternal deaths (0.1%). Preterm and low birthweight infants represented a disproportionate number of stillbirths and pre-discharge deaths, yet very few were born at ≤1500g or <28w. More pre-discharge deaths and stillbirths occurred after maternal referral and with cesarean section. Half of maternal deaths occurred in women who had undergone cesarean section.

**Conclusion:**

Maternity registers are a valuable data source for understanding pregnancy outcomes including those mothers and infants at highest risk of perinatal mortality. Strengthened register data in Kenya and Uganda highlight the need for renewed focus on improving care of preterm and low birthweight infants and expanding access to emergency obstetric care. Registers also permit enumeration of pregnancy loss <28 weeks. Documenting these earlier losses is an important step towards further mortality reduction for the most vulnerable infants.

## Introduction

While maternal mortality decreased 44% globally between 1990 and 2015, a considerable mortality burden persists in low-income regions [1]. Significantly less progress has been made worldwide in neonatal mortality, which accounts for 43% of deaths among children under age five (2.1 million annually) [2]. In addition, there are an estimated 2.6 million third trimester stillbirths globally each year [3], more than doubling the mortality associated with viable pregnancies. Neonatal deaths and stillbirths have been described as the “unfinished agenda” of the Millennium Development goals and the World Health Organization (WHO) Every Newborn Action Plan presents a vision for ending preventable newborn deaths and stillbirths by 2035 [4]. One of the strategic objectives of the plan is to count every birth and its outcome [4]. As facility-based deliveries increase [5], strengthening routine health systems data, including maternity ward birth registers, offers a promising opportunity for more completely and accurately enumerating pregnancy outcomes and highlighting opportunities for perinatal mortality prevention.

Sub-Saharan Africa is an area of particular focus given the tremendous burden of mortality in the region. Maternal mortality estimates remain as high as 546 per 100,000 live births [1], neonatal mortality 25.9 per 1000 live births [2], and stillbirths 28.7 per 1000 total births [3]. Although these figures represent large strides in documenting pregnancy outcomes, underestimation remains a tremendous barrier to perinatal mortality reduction. One key contributor to underestimation is lack of adequate register systems. The Lancet study on civil registration and vital statistics found that, of Sub-Saharan African countries with civil registration and vital statistics data available between 2005 and 2012, only South Africa had a satisfactory register system with the quality being very low in other countries [6]. Other barriers to accurately enumerating pregnancy outcomes include challenges with gestational age estimates, which have varying levels of accuracy depending on type and timing of pregnancy dating (last reported menstrual period, ultrasound assessment, size-based estimates) [7, 8]. Additionally, the inconsistent definition of stillbirth in the literature, with the lower gestational age limit ranging from 18–28 weeks [2], further confounds classification and international comparisons.

Nevertheless, the magnitude of preventable death is large. The leading cause of neonatal mortality is preterm birth [9] and nearly 85% of preterm births occur after 32 weeks gestation [10], when outcomes can be much improved [11]. In fact, estimates suggest that 75% of preterm neonatal deaths could be avoided without neonatal intensive care [12]. Additionally, roughly half of stillbirths occur during the intrapartum period [3], the time period where prevention is most possible.

This study aims to contribute to the global quality improvement and mortality prevention efforts by using strengthened maternity register data from Kenya and Uganda to characterize facility-based pregnancy outcomes including maternal, neonatal, and fetal outcomes at the time of delivery as well as discharge outcomes for live born infants. A second objective of this study is to understand the relationship between pregnancy outcomes and infant, maternal and facility characteristics to inform opportunities for facility-based perinatal mortality prevention.

## Materials and methods

### Study design and setting

This study is a descriptive, cross-sectional analysis of labor ward maternity registers in Kenya and Uganda between October 1^st^, 2016 and March 31^st^, 2018. Data were collected as part of the East Africa Preterm Birth Initiative (PTBi) [13]. This initiative is a partnership between the University of California San Francisco, Kenya Medical Research Institute, University of Rwanda, Rwanda Biomedical Center, and Makerere University in Uganda. In Kenya and Uganda specifically, PTBi is conducting a randomized cluster trial to evaluate the impact of an intrapartum quality improvement package on neonatal survival in preterm and low birthweight infants (clinicaltrials.gov, NCT03112018). The full study protocol is available elsewhere [14]. This cross-sectional analysis includes both control and intervention sites and is not an evaluation of the impact of the trial.

Maternity register data were gathered from 23 health facilities including 17 in Migori county in western Kenya and six in Busoga region in eastern Uganda. In Migori county, facility births represent 53% of all births [15] and in Busoga approximately 77% of deliveries occur in facilities [16]. The facilities included in this analysis were the largest facilities in each location and based on population and reported births, it is estimated that included facilities covered approximately 20–30% of all births in the two regions [17–19].

Within each country the level of care of included facilities varied. However, across both countries facilities ranging from level III through VI were represented [for facility level definitions see references 20, 21]. In Kenya, the 17 facilities included nine level III health centers and eight level IV district referral hospitals. Cesarean sections were performed in level IV facilities only. Two of the 17 Kenyan facilities had newborn special care units. However, only one had a pediatrician on staff. Six facilities had a general doctor, six had a clinical officer, and the remaining five employed nurse midwives only [22].

In Uganda by contrast, all facilities were hospitals including five level V and one level VI facility. All Ugandan facilities were capable of performing cesarean sections and all had newborn special care units. Two hospitals had staff pediatricians and the remainder employed a general doctor [22].

### Data sources and quality

Anonymized patient level delivery data were extracted monthly from maternity registers. Pre-existing national maternity registers were used for this study. However, prior to the study period, data strengthening efforts were completed as part of the PTBi trial to improve the accuracy and completeness of these maternity registers. These efforts included provision of supplies (pregnancy wheels, tape measures, digital scales) with skill building sessions, monthly training and mentoring of labor and delivery staff on standard indicator definitions, and monthly feedback on the completeness of registers. Particular emphasis was placed on the accuracy of gestational age assessments, which were estimated by labor and delivery providers based on reported last menstrual period, fundal height, or antenatal records carried by the mother. Ultrasound was not universally available during antenatal care or at the time of delivery.

The impact of data strengthening on register completeness has been evaluated and full results are available elsewhere [23]. In brief, in Kenya average completion rates increased from 93 to 97% for gestational age, 87 to 98% for birthweight, 97 to 99% for 1-minute APGAR, and 74 to 88% for infant status at discharge from the preliminary assessment to 6 months post data strengthening [23]. In Uganda, average completion rates increased from 52 to 87% for gestational age, 89 to 94% for birthweight, 93 to 96% for 1-minute APGAR, and 86 to 88% for infant status at discharge [23].

Infant, maternal and facility characteristics abstracted from registers and their completeness in this study are as follows: infant sex 91%, multiple gestation 97%, gestational age 86%, birth weight 92%, maternal age 99%, incoming maternal referral status 59% (only available in Uganda), delivery mode 93%, and facility level 100%.

### Pregnancy outcome definitions

Register entries were identified as deliveries if at least one of the following indices was documented: 1-minute Apgar score, birth weight, infant sex, birth outcome, or discharge status. Pregnancy outcomes were then classified as 1) live birth, 2) early stillbirth, 3) late stillbirth, or 4) spontaneous abortion.

Live births were defined in this study as infants born with signs of life (as noted by the health care provider at the time of birth and validated by non-zero 1-minute Apgar score) weighing ≥500 grams or, if no birth weight was recorded, ≥24 weeks completed gestation. This differs from the WHO definition of live birth, which includes any infant born with signs of life regardless of gestational age or birth weight [12]. The definition was chosen in part to permit classification of spontaneous abortions, which were defined as any fetus born weighing <500 grams or, if no birthweight was recorded, <24 weeks gestational age.

Stillbirths were classified as early or late. The WHO definition of stillbirth was used to define late stillbirths in this analysis—infants born without signs of life weighing ≥1000 grams or, if no birth weight was recorded, ≥28 weeks completed gestation [2]. Early stillbirths were defined as infants born without signs of life weighing between 500 and 999 grams or, if no birth weight was recorded, between 24 and 27 weeks completed gestation. Some stillbirths were further identified as fresh (i.e. intrapartum) or macerated based on infant appearance to the provider at the time of delivery, although not a required field in registers. Training was provided on visual differentiation of fresh versus macerated stillbirths but fetal heart tone monitoring was not routinely available in study facilities.

Discharge outcomes were examined for all live born infants. In Kenya, registers included a field for discharge outcome distinct from birth outcome. In Uganda, there was only one field for infant status. In both countries, when delivery and discharge status could not be distinguished or there was conflicting information (i.e. non-zero 1-minute Apgar categorized as a stillbirth), Apgar scores were used to differentiate stillbirths from live births experiencing an immediate neonatal or pre-discharge death. Pre-discharge maternal mortality was also examined and was a unique field in registers in both countries.

### Inclusion/exclusion criteria

Entries excluded from this analysis included 1) births before arrival (n = 606), as the aim was to characterize facility-based outcomes and 2) births with no documented birth weight or gestational age (n = 36), as this prohibited outcome classification. Mothers were excluded if 1) they delivered before arrival (n = 562) or 2) were discharged pregnant (n = 9202). A unique maternal identification code was used to link maternal and neonatal data.

### Statistical analysis

Data are summarized using descriptive frequencies. Pearson chi square test was used to compare pregnancy outcomes by country as well as by maternal, infant, and facility-based co-variates. The Fisher’s exact test was substituted for cases of small sample size (n<5) in instances where models converged. Early stillbirths and spontaneous abortions were excluded from the analyses by birth weight and gestational age as these outcomes were pre-defined by a narrow range of birth weights and gestational ages.

A sub analysis was performed to compare fresh versus macerated late stillbirths. Other analyses available upon request include: country specific analyses and a sub analysis of multiple gestation vs singletons. All analyses except Fisher’s exact tests were performed using SPSS 23 [24]. Fisher’s exact tests were performed in STATA 14 [25].

### Ethical considerations

This study was approved by Institutional Review Board at the University of California San Francisco (Study no: 16–19162), the Kenyan Medical Institute Scientific and Ethics Review Unit (SERU protocol no: KEMRI/SERU/CCR/0034/3251), the Makerere University Higher Degrees, Research, and Ethics Committee (Protocol ID: IRB00011353), and the Uganda National Council of Science and Technology. There was a waiver of consent to obtain line-item level data from maternity registers.

### Patient and public involvement

De-identified data were collected from maternity registers, so there was no direct patient contact or time spent for this analysis. Results will be disseminated to health workers and health authorities from research areas.

## Results

A total of 50,981 births to 48,675 mothers were included. Two thirds of deliveries were in Uganda (n = 34,015) and one third in Kenya (n = 16,966).

### Maternal discharge outcomes

There were 64 documented pre-discharge maternal deaths over the study period (approximately 100 pre-discharge deaths per 100,000 total births), 20 in Kenya and 44 in Uganda. Half of the mothers who died had cesarean sections and the remainder had vaginal deliveries. Further, half of the mothers who died in childbirth had liveborn infants. Of the other half, 27 had late stillbirths, one had an early stillbirth, and three had spontaneous abortions. Another 11% of mothers who delivered in study facilities (n = 5344) left prior to discharge, were transferred, or had an undocumented discharge status (Table 1).

**Table 1:** Pregnancy and discharge outcomes amongst all facility-based deliveries in Kenya and Uganda over an 18-month period (N = 48,675 mothers and 50,981 infants).

Outcome	Total	Kenya	Uganda
	n (% column)		
**Pregnancy Outcome**			
Live birth^1^	46531 (91.3)	16363 (96.4)	30168 (88.7)
Late stillbirth^2^	1834 (3.6)	402 (2.4)	1432 (4.2)
Early stillbirth^3^	244 (0.5)	54 (0.3)	190 (0.6)
Spontaneous abortion^4^	2372 (4.7)	147 (0.9)	2225 (6.5)
**Neonatal Discharge Outcome**^5^			
Discharged alive	40972 (98.4)	14396 (98.9)	26576 (98.2)
Pre-discharge death	653 (1.6)	158 (1.1)	495 (1.8)
**Maternal Discharge Outcome**			
Discharged alive	43267 (88.9)	14902 (95.9)	28365(85.6)
Maternal mortality	64 (0.1)	20 (0.1)	44 (0.1)
Other^6^	5344 (11.0)	616 (4.0)	4728 (14.3)

1. Live birth = infant born weighing ≥500 grams or, if no birth weight recorded, at ≥24 weeks gestational age

2. Late stillbirth = ≥1000 grams, or if no birth weight recorded, ≥28 weeks gestational age

3. Early stillbirth = 500–999 grams, or if no birth weight recorded, 24–27 weeks gestational age

4. Spontaneous abortion = infants born at <500 grams or, if no birth weight recorded, <24 weeks gestational age

5. Discharge outcomes among live born infants

6. Includes mothers who left prior to discharge, were transferred to another facility (i.e. referred out after delivery), or for whom discharge status was undocumented

### Pregnancy outcomes

**Fig 1:**
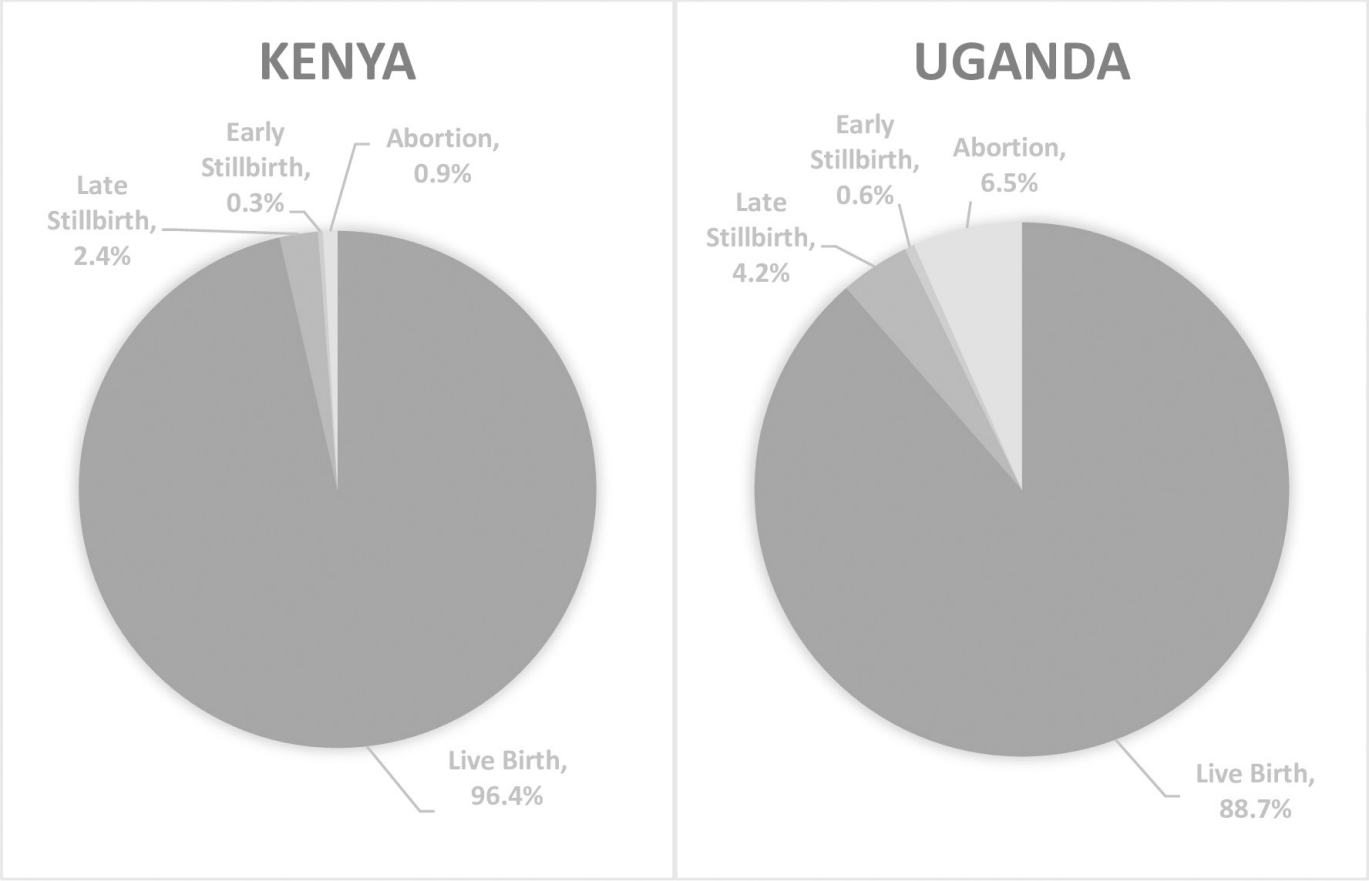
Pregnancy outcomes by country (Kenya n = 16,966, Uganda n = 34,015).

Of all registered deliveries, 91.3% were live births, 4.1% stillbirths (0.5% early and 3.6% late), and 4.7% spontaneous abortions. In Uganda, 88.7% were live births, 0.6% early stillbirths, 4.2% late stillbirths, and 6.5% spontaneous abortions. In Kenya, 96.4% were live births, 0.3% early stillbirths, 2.4% late stillbirths, and 0.9% spontaneous abortions (Table 1, Fig 1, *p*<0.01).

Pregnancy outcomes varied by gestational age, gestation type (i.e., single or multiple), and birth weight as well as by maternal age, maternal referral status, delivery mode, and facility level (Table 2, all *p*<0.01). Over 85% of live births occurred at term. The preterm birth rate was 134 per 1000 live births (148/1000 in Kenya and 126/1000 in Uganda). Very few live births occurred at <28 weeks gestation (0.5%), with increasing numbers from 28 to 32 weeks (3.1%) and 33 to 36 weeks (9.8%). For late stillbirths, the percentages were 3.1%, 17.3%, and 16.5% by gestational age grouping respectively. Approximately 38% of late stillborn infants weighed ≤2500 grams (11% ≤ 1500g), whereas only 13% of live births were low birthweight. The prevalence of multiple gestation pregnancies were roughly equivalent in live births (4.1%), early stillbirths (5.1%), and late stillbirths (4.9%) but lower in spontaneous abortions (0.7%).

**Table 2:** Infant, maternal, and facility characteristics associated with registered pregnancy outcomes in Kenya and Uganda (N = 50,981).

Characteristic	N	Live Birth^1^ (N = 46531)	Late Stillbirth^2^ (N = 1834)	Early Stillbirth^3^ (N = 244)	Spontaneous Abortion^4^ (N = 2372)	
		n (% column)				*p*-value^5^
**Infant Characteristics**						
**Sex**						
Male	46533	23054 (51.6)	861 (52.0)	59 (53.6)	39 (53.4)	0.94
Female		21640 (48.4)	795 (48.0)	51 (46.4)	34 (46.6)	
**Multiple gestation**						
Yes	49627	1850 (4.1)	88 (4.9)	12 (5.1)	17 (0.7)	<0.01
No		43390 (95.9)	1698 (95.1)	223 (94.9)	2349 (99.3)	
**Gestational age in weeks**						
≤ 27	42355	218 (0.5)	50 (3.1)			<0.01
28–32		1271 (3.1)	279 (17.3)			
33–36		3989 (9.8)	265 (16.5)			
37–40		32801 (80.5)	964 (59.9)			
≥ 41		2467 (6.1)	51 (3.2)			
**Birth weight in grams**						
≤ 500	46600	9 (0.0)	0 (0.0)			<0.01**
501–1500		706 (1.6)	181 (11.0)			
1501–2500		5239 (11.7)	441 (26.9)			
2501–3500		30480 (67.8)	772 (47.0)			
3501–4500		8326 (18.5)	225 (13.7)			
>4500		199 (0.4)	22 (1.3)			
**Maternal Characteristics**						
**Age in years**						
≤19	50553	9740 (21.1)	327 (18.0)	58 (24.1)	428 (18.4)	<0.01
20–24		16225 (35.1)	551 (30.4)	87 (36.1)	641 (27.5)	
25–29		10728 (23.2)	423 (23.3)	45 (18.7)	536 (23.0)	
30–34		6187 (13.4)	321 (17.7)	37 (15.4)	368 (15.8)	
≥35		3285 (7.1)	193 (10.6)	14 (5.8)	358 (15.4)	
**Incoming referral status**^6^						
Yes	30313	3242 (11.9)	342 (26.0)	17 (10.2)	92 (6.0)	<0.01
No		24061 (88.1)	971 (74.0)	150 (89.8)	1438 (94.0)	
**Delivery mode**						
Vaginal	47202	36330 (80.0)	1149 (69.9)	71 (92.2)	71 (95.9)	<0.01*
Caesarean Section		9077 (20.0)	495 (30.1)	6 (7.8)	3 (4.1)	
**Facility Characteristic**						
**Level**						
III	50981	4515 (9.7)	54 (2.9)	4 (1.6)	32 (1.3)	<0.01**
IV		11848 (25.5)	348 (19.0)	49 (20.2)	115 (4.8)	
V		21958 (47.2)	1067 (58.2)	180 (74.1)	2200 (92.7)	
VI		8210 (17.6)	365 (19.9)	10 (4.1)	25 (1.1)	

1. Live birth = infant born weighing ≥500 grams or, if no birth weight recorded, at ≥24 weeks gestational age

2. Late stillbirth = ≥1000 grams, or if no birth weight recorded, ≥28 weeks gestational age

3. Early stillbirth = 500–999 grams, or if no birth weight recorded, 24–27 weeks gestational age

4. Spontaneous abortion = infants born at <500 grams or, if no birth weight recorded, <24 weeks gestational age

5. Statistical test used. If not otherwise marked, the Pearson’s chi-square test was used. If marked with *, Fisher’s Exact Test was used. If marked with ** the Pearson’s chi-square test was used despite small sample size due to issues with model convergence.

6. Maternal referral status into study facilities is only available in Uganda

With respect to maternal and facility co-variates, the majority of deliveries occurred at level IV and V facilities. However, the spread across higher and lower level facilities was greater for live births and late stillbirths compared to early stillbirths and spontaneous abortions. Over a quarter of late stillborn infants were born after maternal referral into study facilities (26%). By comparison, only 11.9% of live births were following maternal referral, 10.2% of early stillbirths, and 6% of spontaneous abortions. Additionally, 30% of late stillborn infants were delivered by cesarean section compared to 20% of live born infants. Six early stillbirths and three spontaneous abortions were delivered via cesarean section (the majority of these were twin deliveries or associated with extenuating circumstances like uterine rupture or fetal anomaly). The percentage of deliveries via cesarean section after incoming maternal referral was 46.8% for live births, 44.7% for late stillbirths, and 0% for early stillbirths and spontaneous abortions.

A sub-analysis of late stillbirth type revealed 46% of late stillbirths were categorized as fresh (n = 850), 33% as macerated (n = 612), and the remainder were undifferentiated (n = 372). Gestational age, birth weight, maternal referral status, delivery mode, and facility level differed by type of late stillbirth (Table 3, maternal referral *p* = 0.02, all others *p*<0.01). Conversely, infant sex, single versus multiple gestation, and maternal age did not differ by stillbirth type. More fresh stillbirths were born after incoming maternal referral (31.8%) compared to macerated stillbirths (24.8%). Approximately 40% of fresh stillbirths were delivered by cesarean section compared to 21.8% of macerated stillbirths. Roughly 70% of fresh stillbirths were born at term compared to 58% of macerated stillbirths. A slightly higher percentage of macerated (3.5%) compared to fresh (2.7%) stillbirths occurred at ≥41 weeks. Finally, more macerate stillbirths were low birthweight (45.6%) compared to fresh stillbirths (31%).

**Table 3:** Infant, maternal, and facility characteristics associated with fresh versus macerated late stillbirths in Kenya and Uganda (N = 1462).

	Late Stillbirth^1^			
		Fresh Stillbirth	Macerated Stillbirth (N = 612)	
		(N = 850)		
		n (% column)		*p*-value^2^
**Infant Characteristics**				
**Sex**				
Male	1377	421 (52.2)	290 (50.9)	0.66
Female		386 (47.8)	280 (49.1)	
**Multiple gestation**				
Yes	1422	42 (5.1)	31 (5.2)	0.9
No		787 (94.9)	562 (94.8)	
**Gestational age in weeks**				
≤ 27	1280	7 (0.9)	25 (4.7)	<0.01
28–32		91 (12.2)	103 (19.2)	
33–36		115 (15.5)	97 (18.1)	
37–40		511 (68.7)	292 (54.5)	
≥ 41		20 (2.7)	19 (3.5)	
**Birth weight in grams**				
≤ 500	1362	0 (0.0)	0 (0.0)	<0.01*
501–1500		67 (8.4)	79 (14.0)	
1501–2500		180 (22.6)	179 (31.6)	
2501–3500		416 (52.3)	230 (40.6)	
3501–4500		121 (15.2)	71 (12.5)	
>4500		12 (1.5)	7 (1.2)	
**Maternal Characteristics**				
**Age in years**				
≤19	1451	150 (17.8)	118 (19.4)	0.88
20–24		250 (29.7)	179 (29.4)	
25–29		207 (24.6)	138 (22.7)	
30–34		146 (17.3)	105 (17.2)	
≥35		89 (10.6)	69 (11.3)	
**Incoming referral status**^3^				
Yes	1011	202 (31.8)	93 (24.8)	0.02
No		434 (68.2)	282 (75.2)	
**Delivery Mode**				
Vaginal	1364	471 (59.6)	449 (78.2)	<0.01
Caesarean Section		319 (40.4)	125 (21.8)	
**Facility Characteristic**				
**Level**				
III	1462	22 (2.6)	25 (4.1)	<0.01
IV		152 (17.9)	171 (27.9)	
V		503 (59.2)	285 (46.6)	
VI		173 (20.4)	131 (21.4)	

1. Late stillbirth = ≥1000 grams, or if no birth weight recorded, ≥28 weeks gestational age

2. Statistical test used. If not otherwise marked, the Pearson’s chi-square test was used. If marked with *, Fisher’s Exact Test was used.

3. Maternal referral status into study facilities is only available in Uganda

### Neonatal discharge outcomes

Of live born infants, 1.6% (n = 653) died prior to discharge. The pre-discharge mortality rate was higher in Uganda (18/1000) than Kenya (11/1000, Table 1, *p*<0.01). The overall pre-discharge neonatal mortality rate was 16 per 1000 live births.

Discharge status was unknown for 4,906 infants (10.5% of live births). This may include infants referred out to higher level facilities. Outgoing infant referral status was not recorded in registers. Of infants with no documented discharge status, 19.6% were preterm, 17.9% were low birthweight, 4.6% multiple gestations, 7.6% were born to mothers referred into study facilities prior to delivery, and 21.9% were born via cesarean section. For infants with known discharge status, outcomes varied by all infant, maternal, and facility characteristics analyzed (Table 4, all *p*≤0.01).

**Table 4:** Infant, maternal, and facility characteristics associated with neonatal discharge outcomes in Kenya and Uganda (N = 41,625).

Characteristic	N	Discharged Alive (N = 40972)	Pre-Discharge Death (N = 653)	
		n (% column)		*p*-value^1^
**Infant Characteristics**				
**Sex**				
Male	40289	20467 (51.6)	352 (56.6)	0.01
Female		19200 (48.4)	270 (43.4)	
**Multiple gestation**				
Yes	40546	1563 (3.9)	72 (11.1)	<0.01
No		38337 (96.1)	574 (88.9)	
**Gestational age in weeks**				
≤ 27	36954	129 (0.4)	42 (7.3)	<0.01
28–32		926 (2.5)	128 (22.1)	
33–36		3438 (9.5)	91 (15.7)	
37–40		29663 (81.5)	294 (50.8)	
≥ 41		2219 (6.1)	24 (4.1)	
**Birth weight in grams**				
≤ 500	40584	5 (0.0)	11 (1.8)	<0.01**
501–1500		386 (1.0)	157 (25.4)	
1501–2500		4477 (11.2)	143 (23.1)	
2501–3500		27371 (68.5)	237 (38.3)	
3501–4500		7551 (18.9)	67 (10.8)	
>4500		176 (0.4)	3 (0.5)	
**Maternal Characteristics**				
**Age in years**				
≤19	41347	8496 (20.9)	163 (24.8)	<0.01
20–24		14322 (35.2)	204 (31.1)	
25–29		9506 (23.4)	129 (19.6)	
30–34		5461 (13.4)	104 (15.8)	
≥35		2905 (7.1)	57 (8.7)	
**Incoming referral status**^2^				
Yes	24956	2955 (12.1)	109 (22.7)	<0.01
No		21521 (87.9)	371 (77.3)	
**Delivery mode**				
Vaginal	40892	32397 (80.5)	408 (64.8)	<0.01
Cesarean Section		7865 (19.5)	222 (35.2)	
**Facility Characteristic**				
**Level**				
III	41659	4254 (10.4)	27 (4.1)	<0.01
IV		10148 (24.8)	136 (20.5)	
V		19565 (47.7)	396 (59.6)	
VI		7028 (17.1)	105 (15.8)	

1. Statistical test used. If not otherwise marked, the Pearson’s chi-square test was used. If marked with ** the Pearson’s chi-square test was used despite small sample size due to issues with model convergence.

2. Maternal referral status into study facilities is only available in Uganda

The majority of pre-discharge deaths occurred at level V facilities (59.6%) and very few occurred at level III facilities (4.1%). A higher percentage of infants who died prior to discharge were born to women ≤19 or ≥35 (33.5%) years old compared with infants discharged alive (28%). Just under a quarter (22.7%) of infants dying before discharge were born after maternal referral. By comparison, of infants surviving to discharge, only 12.1% were born after maternal referral. Over one third of infants (35.2%) who died before discharge were born via caesarean section compared to 19.5% of those discharged alive.

More pre-discharge deaths were male (56.6%) than female (43.4%). Eleven percent of pre-discharge deaths were multiple gestation infants compared to only 3.9% of infants discharged alive. A higher percentage of infants deceased at discharge were preterm (45.1%) compared to those alive at discharge (12.4%). Of those infants that survived to discharge, 9.5% were born between 33 and 36 completed weeks of gestation, 2.5% between 28 and 32 weeks, and 0.4% <28 weeks. Approximately 12% of infants discharged alive were low birthweight, with nearly all of those infants falling in the 1501 to 2500-gram range. Half of pre-discharge deaths (50.3%) were low birthweight infants.

## Discussion

This study used strengthened maternity register data to characterize pregnancy outcomes for nearly 51 thousand deliveries from 23 health care facilities in Kenya and Uganda over 18 months including pregnancy loss at <1000g or 28 weeks. Results highlight three priorities for further facility-based perinatal mortality reduction efforts: 1) continued attention to complete and accurate documentation of all pregnancy and discharge outcomes, 2) improvement in the care of low birth weight and preterm infants, and 3) expanded access to high quality emergency obstetric and neonatal care.

### I. Complete and accurate documentation of all pregnancy and discharge outcomes

Neonatal mortality and stillbirths occur within the same continuum of care, but are nevertheless often studied and reported separately leading to an underappreciation of the mortality burden associated with viable pregnancies. Further, pregnancy loss at <28 weeks is often excluded from international statistics with the current WHO definition of stillbirth [2]. This study provides a unique cross-sectional assessment of both pregnancy and neonatal discharge outcomes including pregnancy loss, early and late stillbirth, and pre-discharge neonatal deaths underscoring the value of register data.

#### Pre-discharge neonatal deaths

The pre-discharge, facility-based neonatal death rate in this study was 16 per 1000 live births, substantially lower than the published regional estimate for overall population-based 28-day neonatal mortality in sub-Saharan Africa (25.9 per 1000 live births) [2]. By comparison, the Kenya Demographic Health Survey (DHS) in 2014, reported a neonatal mortality rate of 22 (and one of 19 per 1000 in Nyanza region where Migori County is located) [26], and the Uganda DHS reported a neonatal mortality rate of 27 per 1000 nationally (28 per 1000 in Busoga region) [16]. A recent systematic review found that approximately two thirds of neonatal deaths in LMIC settings occur between day zero and two of life [27]. Further, a multi-country analysis reported the average maternal postpartum stay is 2 days in Kenya and 1.4 days in Uganda for singleton, vaginal deliveries [28]. Therefore, based on timing alone, the pre-discharge neonatal mortality rate in this study likely underestimates the overall 28-day neonatal morality rate by at least one third. With this adjustment, the neonatal mortality rate in this study would rise to approximately 24, comparable to regional estimates [2].

Although with this adjustment rates in this study are comparable to current regional estimates, it is important to acknowledge that rates in this study are facility-based rates and the true population-based rates are likely higher. Additionally, some study characteristics suggest underreporting and emphasize the need for continued attention to both complete and accurate documentation of early neonatal deaths. First, discharge status was unknown for 4,906 infants (10.5%) in this study. Of these infants, nearly 20% were preterm and 18% low birthweight. Second, outgoing infant referral status was not known nor the ultimate discharge outcome for any infants referred to higher levels of care. Thus, ongoing efforts focused on documentation would likely only reveal more potentially preventable deaths.

#### Late stillbirths

The late stillbirth rate in this study was 36 per 1000 total births. This is significantly higher than the previously published stillbirth estimate for sub-Saharan Africa of 29 per 1000 total births [3]. The Uganda DHS reports 16 stillbirths per 1000 pregnancies reaching 7 completed months [16], which equates to late stillbirths in this study; whereas Kenya DHS reported just 13.3 per 1000 pregnancies reaching 7 months [26].

There is good reason to believe previously reported regional stillbirth rates may be significant underestimates. The first published global estimates of stillbirths emerged as recently as 2011 [29] and the 2016 Lancet series on stillbirths estimated that less than 5% of stillbirths are documented globally [3]. Further, with the data strengthening efforts in this study as well as the ability to use a registered non-zero one-minute APGAR score to distinguish stillbirth from early neonatal death, we believe the reported higher stillbirth rate in this study may more accurately reflect the true facility-based incidence. The chance to train facility-based staff on standard indicator definitions and to use data triangulation to validate outcomes, highlights the opportunity register data may afford to more accurate depict the significant need for stillbirth prevention. APGAR scores are only one possibility for data triangulation and other options may include fetal heart rate monitoring, where available.

Of particular interest is improving accuracy of classification of fresh versus macerated stillbirths. While training was provided to labor and delivery providers as part of the data strengthening efforts in this study, about 20% of late stillbirths were not classified as fresh or macerated. Fetal heart rate assessment via doppler is one tool that has been successfully employed in Tanzania [30]. Requiring this documentation and reducing stigma around reporting fresh stillbirths are other important steps that may help improve the completeness and accuracy of this documentation and thus help focus mortality reduction efforts on preventable deaths.

#### Early stillbirths and spontaneous abortions

Of all registered pregnancy outcomes, 0.5% were early stillbirths and 4.7% spontaneous abortions. Neither of these outcomes are routinely measured in LMIC settings and their documentation is a unique contribution of this study despite likely significant underestimation. Studies from high-income settings, suggest that approximately 17% of stillbirths occur between 24 and 28 weeks [31] and that between 10–15% of pregnancies end in early fetal death before 24 weeks [32].

Underestimation stems from challenges associated with accurately measuring pregnancy loss including dating accuracy [7, 8] and location of delivery. Specifically, records from gynecology wards were not included in this study. However, many women with early pregnancy loss were likely triaged to the gynecology ward. In addition, early spontaneous abortion and stillbirth may disproportionately occur at home, and uncomplicated cases may not present for institutional care in settings such as Uganda and Kenya, where approximately a third of women deliver at home [16, 26].

Despite the challenges, documentation of these pregnancy losses is important. In high income countries the decline in early stillbirth rates is similar to that of late stillbirths internationally [32]. This suggests that with continued improvements in technology and access to care, the survival of infants <28 weeks in LMIC settings will be attainable in the future. In fact, in this study, 129 infants born at ≤27 weeks survived to discharge. Enumerating these deliveries is the first step towards advancing care.

### II. Improvement in the care of low birth weight and preterm infants

Of live births in this study, 13.4% were preterm births and 13.3% were low birthweight infants. This is comparable to regional estimates (12% and 13% respectively) [10, 33]. Despite making up a relatively low percentage of live births, preterm and low birthweight infants disproportionately accounted for nearly half of pre-discharge deaths. Nevertheless, only 4% of preterm infants in this study were born extremely premature (<28 weeks) and further, only 12% of low birthweight infants were very low birth weight (≤1500g). In sum, this is consistent with the Global Action Report on Preterm Birth’s conclusion that 75% of preterm deaths are preventable without intensive care [12]. Simple, low-cost measures for averting these deaths may include Kangaroo Mother Care, appropriate administration of antenatal corticosteroids, hygienic cord care, and exclusive breast-feeding support [34].

It has been suggested that preterm stillbirths should be included in reporting preterm birth rates to more accurately reflect international disparities, partially due to common misclassification of early neonatal deaths as stillbirths in preterm infants in low income settings [9]. Maternity registers afford this opportunity, especially with data strengthening around outcome definitions. In this study, 37% of late stillbirths were preterm deliveries. Nearly a third (29%) of these preterm stillbirths were fresh stillbirths. Fresh stillbirths are perhaps the most likely to be misclassified and represent another group that stands to benefit from increased efforts to improve the care of small and preterm infants immediately after birth.

### III. Expanded access to high quality emergency obstetric and neonatal care

Intrapartum-related events are the second leading cause of early neonatal mortality globally [9] and are directly associated with access to emergency obstetric and neonatal care [35, 36]. Maternal referral and delivery via cesarean section serve as proxies for access to care and maternity registers offer the opportunity to look at these two indicators together. Notably, in this study there were more cesarean sections following incoming maternal referral compared to overall for both live births (47% versus 20% of deliveries) and late stillbirths (45% versus 30% of deliveries). Additionally, of neonatal deaths, 23% occurred after maternal referral and 35% occurred with cesarean section. Of late stillbirths, 26% occurred after maternal referral and 30% occurred with cesarean section. Looking only at fresh stillbirths, 32% occurred after maternal referral and 40% with cesarean section. Finally, 75% of neonatal deaths and 78% of stillbirths occurred at level V and VI referral facilities. In sum, this data suggest pregnancies with intrapartum complications are being referred to higher levels of care. However, the higher likelihood of cesarean section after referral and the disproportionately high stillbirth and neonatal mortality rates after maternal referral and with cesarean section suggest a lack of access to and/or delays in emergency obstetric care and neonatal resuscitation. Improving the efficiency of identification and referrals is important and the need for improved skills for safe cesarean section also cannot be excluded.

Emergency obstetric care is critical for not only neonatal survival but also for maternal survival. Nearly 70% of maternal deaths occur prior to, during, or shortly after birth [37]. The maternal, facility-based mortality rate in this study was approximately 100 per 100,000 total births, well below regional population-based estimates of 546 per 100,000 live births [38] and national estimates in Kenya of 362 per 100,000 births [26] and Uganda of 336 per 100,000 births [16]. This may indicate underreporting as 11% of mothers had unknown discharge status, but it may also indicate maternal deaths are more often occurring at home or en route to facilities. Half of the observed maternal deaths followed caesarean section, which is consistent with other reports of the significant risk associated with caesarean section in sub-Saharan Africa [39].

#### Study strengths and limitations

This study has many strengths including the large sample size, two country setting including a broad range of facility levels, data strengthening efforts, concurrent analysis of both delivery and discharge outcomes, and extension of gestational age limits to more comprehensively document spontaneous abortion and early stillbirth. However, this study also has important limitations.

#### Country differences

Uniformly, mortality rates were higher in Uganda compared to Kenya in this study. These differences are likely attributable to facility characteristics. All included Ugandan study facilities were level V and VI hospitals whereas Kenyan study facilities were level III or IV. Assuming complicated pregnancies are referred to higher levels of care, infants born at the study facilities in Kenya may have represented lower risk pregnancies, whereas in Ugandan facilities, where 12.2% of all births occurred after incoming maternal referral, there was likely a higher prevalence of high-risk pregnancies. More research is needed to rule out other possibilities such as differences in quality of care and other causes of morbidity that could be different in Uganda and Kenya. While direct comparison of countries is not possible due to these facility level differences, when taken in sum, the spread of facilities in this study fairly represents the various tiers of care in the region and improves the generalizability of the conclusions.

#### Data quality and generalizability

Despite the benefits of using maternity register data highlighted throughout the paper, there are also important drawbacks. Data were recorded by frontline providers and were not 100% complete. Efforts to limit observer bias included explicit provider instruction on standard indicator definitions during data strengthening efforts. Data were also cleaned (for example, one-minute APGAR scores were used to validate live birth and stillbirth diagnoses). Due to known issues with gestational age estimates in low income settings [7, 8], we chose to primarily define birth outcome based on weight and, only if weight was missing, gestational age was used. Nevertheless, reported gestational age serves as the foundation for preterm birth rate estimates.

This study seeks to characterize facility-based pregnancy outcomes and thus is not generalizable to the proportion of the population delivering outside of facilities, including at home. Additionally, some facilities included in this study were part of the PTBi trial. This study was not an evaluation of the PTBi trial and includes both intervention and control sites. However, mortality rates may be decreased as a result of the study. Nevertheless, overall concordance with regional estimates suggest the impact of this bias is likely limited.

#### Other limitations

Other important limitations to discuss include lack of data on congenital anomalies. Anomalies are a significant cause of neonatal mortality and morbidity, yet were not systematically recorded in this sample. Additionally, prenatal care, while important, is not captured in registers. Further, spontaneous abortion and early pregnancy loss is likely underestimated given only obstetric registers are included in this analysis. Finally, this study is a cross-sectional analysis of pregnancy and discharge outcomes by infant, maternal, and facility characteristics. It does not adjust for confounding variables and does not attempt to make inferences on causes of perinatal or pre-discharge mortality.

#### Future directions

Translating strengthened registry data and the opportunities this descriptive data highlight for perinatal mortality reduction into concrete action is a crucial next step. At a national level, such data may inform funding priorities for government facilities or systems level improvements, such as referral systems. At a facility level, data may inform maternal and perinatal death surveillance and response (MPDSR) teams [40] or quality improvement projects. Input of strengthened data into the BABIES matrix [41] for risk stratification may also inform quality of care improvement efforts going forward.

## Conclusion

There is a significant unsolved burden of fetal, neonatal, and maternal mortality amongst facility-based deliveries in Kenya and Uganda. Some of the most vulnerable lives include those of preterm and low birthweight infants as well as those pregnancies requiring emergency intrapartum care. Documenting all pregnancy outcomes, including pregnancy loss <28 weeks, to better understand when deaths occur is a critical first step towards highlighting the need for improved care for the most vulnerable infants and pregnancies. As facility-based deliveries increase, maternity registers are valuable data source, particularly with prior attention to data quality and efforts to ensure every pregnancy is counted.
